# Structural and Functional Aspects of DHPM-Thiones and Their Derivatives: A Critical Review of Pharmaceutical Potential

**DOI:** 10.3390/ph19020306

**Published:** 2026-02-12

**Authors:** Artyom Savelyev, Dmitriy Khrustalev, Irina Losseva, Azamat Yedrissov, Anastassiya Khrustaleva, Shapovalenko Sofiya, Marlen Kiikbayev, Rusyaeva Polina, Kazantsev Vladimir

**Affiliations:** School of Pharmacy, NPJSC “Karaganda Medical University”, 40 Gogol St., Karaganda 100008, Kazakhstan; savelev@qmu.kz (A.S.); loseva@qmu.kz (I.L.);

**Keywords:** dihydropyrimidines, DHPM-thiones, structure–activity relationship, microwave flow synthesis, preclinical potential

## Abstract

**Background**: Amidst escalating global challenges such as antimicrobial resistance and post-COVID therapeutic gaps, dihydropyrimidines (DHPs) and their thione derivatives have emerged as a highly promising scaffold for drug development. This systematic review aims to consolidate recent advancements (2020–2025) and evaluate the synthetic innovation, structure–activity relationships (SAR), and preclinical potential of these compounds. **Methods**: A systematic review was conducted according to PRISMA guidelines, searching multiple electronic databases (Scopus, PubMed, Web of Science). Sixty original studies from 2020 to 2025 meeting predefined inclusion criteria were selected for data extraction and qualitative synthesis. **Results**: The analysis reveals a surge in publications (over 300% since 2020). Key structural modifications, such as N-methylation to improve bioavailability and specific substitutions at C4/C5 positions, significantly enhance biological potency, yielding strong inhibitory effects against viral proteases and cancer cell lines. Notable compounds include the apoptosis inducer LaSOM 65 and multitarget Ru(II)–Biginelli hybrids. **Conclusions**: This review affirms the timeliness and translational potential of the DHP scaffold. The field shows bright prospects for advancing to phase I trials by 2030, urging intensified exploration to unlock novel pharmaceuticals from this versatile chemotype.

## 1. Introduction

This section establishes the foundational relevance of 3,4-dihydropyrimidine-2(1H)-thiones (DHPM-thiones), positioning them within the critical context of sulfur-containing heterocycles exemplified by thiazoles. We will discuss their evolving synthetic paradigms, contemporary research trends that reveal a surge in interest, and the methodological framework of this systematic review. By examining both the established promise and identified research gaps, this introduction provides the essential context to appreciate the subsequent detailed analysis of their structure–activity relationships and translational potential.

Global context and relevance of heterocyclic compounds in drug development, with focus on dhpm-thiones and their relation to thiazoles.

SAR analyses have demonstrated that N-methylation enhances bioavailability, while C_4_-fluoroaryl and C_5_-cyano substitutions yield IC_50_ values < 2 μM against SARS-CoV-2 Mpro, PEDV, and glioblastoma [1,2,3]. Prominent candidates, including monastrol-derived LaSOM 65 (apoptosis inducer with LD50 > 2000 mg/kg) and Ru(II)-Biginelli hybrids (dual antiviral–anticancer efficacy), underscore the translational viability of DHPMs [1,4,5]. These findings highlight the broader significance of heterocyclic compounds, particularly those containing sulfur and nitrogen, which have become foundational elements in pharmaceutical development, accounting for over 60% of approved small-molecule drugs, according to FDA database analyses [6,7]. Thiazoles, a prominent sulfur-containing heterocyclic class, exemplify this trend with established applications in antimicrobial (e.g., ceftriaxone), anti-inflammatory (e.g., meloxicam), and antifungal therapies [5,8]. Recent advancements have expanded thiazoles into multitarget agents, such as benzothiazole derivatives for anti-tuberculosis activity, where they inhibit key enzymes with MIC values < 5 μg/mL in ongoing phase II trials [9].

Their structural similarity to dihydropyrimidines (DHPs) and 3,4-dihydropyrimidin-2(1H)-thiones (DHPM-thiones), which share sulfur functionalities and hybridizable scaffolds, facilitates synergistic designs, as seen in thiazole–pyrimidinone fusions that enhance bioavailability and address resistance [10,11,12]. This interconnectedness underscores the broader relevance of these classes amid global challenges, including antimicrobial resistance (causing 1.27 million annual deaths per WHO) and rising cancer incidence (projected 60% increase by 2040) [13,14]. DHPM-thiones, thione analogs of DHPs, build on this by offering superior lipophilicity and chelation properties, making them highly suitable for multi-target drug discovery in the post-COVID landscape [15,16].

Background on DHPM-thiones: Synthesis and structural features.

The Biginelli reaction remains the primary route for DHPM-thione synthesis, with innovations from 2020 to 2025 emphasizing green, scalable methods that parallel thiazole developments [16,17]. Asymmetric variants enable stereoselective induction [18], whereas multitarget ligands incorporate Ca^2+^ blocking, cholinesterase inhibition, and Nrf2 activation [19]. Thione-inclusive scaffolds via Biginelli-like reactions highlight structural parallels to thiazoles and biodegradable polyesters from Biginelli synthesis extend to biomaterials [20]. Modified reactions produce Eg5 inhibitors with in vitro/in vivo anticancer effects [21], and reaction DHPM-thiones with green catalysts target α-amylase and glucosidase [22].

Benzothiazole hybrids are advanced anti-TB compounds that illustrate class overlap [9], while DHPM scaffolds serve diverse therapeutic targets [6]. N_1_-position studies generate bioactive derivatives [23], and mechanistic corrections address the solvent effects and applications [24]. Adenosine A2B antagonists from Biginelli scaffolds exhibit structure–activity relationships (SAR) for colorectal anticancer activity [25], and DHPs function as biotoxic agents on bacterial membranes [26]. ZnO-carbomer gels enhance antibacterial wound dressings [27], and bismuth triflate catalyzes ethyl carboxylates with antioxidant activities [15].

Recyclable iron oxide nanocatalysts yield antimicrobials [11], and MCRs support strategies for Alzheimer’s disease [28]. Microwave MCRs in heterocycle synthesis [16] produce benzyl oxyphenylpyrimidine nitriles that act as apoptotic agents [13].

The structural features of DHPM-thiones, including the C=S group, enable enhanced lipophilicity compared to oxo-analogs, with thiazole-like sulfur enhancing the hybrid potential [5]. Biginelli scaffolds promote wound healing in tilapia gill lines [29], and DES-synthesized DHPs target neuroregeneration [30]. Benzyl sulfonamide-decorated thiones profile carbonic anhydrase with antiproliferative activity [31], while green synthesis yields antitumor DHPs [32]. Pyrimidine derivatives block calcium channels [15], and MCRs surge as synthesis alternatives [7].

Actuality of DHPs and DHPM-thiones: Publication trends and research gaps.

Publication trends from Scopus and PubMed (13 November 2025) show >200 articles on “Biginelli reaction” and “dihydropyrimidin-thiones” from 2020 to 2025, a 300% rise driven by COVID-19 demands [6,7]. Functionalized aminodihydropyrimidines exhibit antibacterial SAR [4], and thiazole-linked pyrimidinones undergo DFT/docking for corrosion and bioactivity [5]. Adamantane-containing DHPs act as antitumor agents [8], and fatty-acid DHPs target breast/gastric cancer [9]. Oxadiazole hybrids inhibit cholinesterase with SAR/in silico [13], pyrazoles as bone anabolics [14], and new DHPs induce cytotoxicity/apoptosis via docking/MD simulations [33].

Indole-DHPs treat visceral leishmaniasis with SAR/mechanism [29], and DHPs combat tuberculosis via docking/MD studies [34]. Dual EGFR/TrkA inhibition unveils antitumor potential [35], nifedipine/monastrol analogs offer antileishmanial/antimicrobial effects [32], and new DHPs inhibit EGFR/HER2. L-asparagine-EDTA nanoparticles catalyze green DHP synthesis [17], and in silico docking targets antimicrobials [25]. Fluorescent DHP hybrids induce cell cycle arrest via the activation of Aurora kinase [26].

Gaps include underemphasis on thione-thiazole hybrids versus standalone thiazoles [5,18,36], with abundant preclinical data (e.g., ADMET for LaSOM 65 [4]) but clinical lags. This review addresses these issues by evaluating the SAR for pharmaceutical prospects and partially integrating thiazole relationships to highlight class synergies.

## 2. Materials and Methods

This systematic review was conducted and reported in accordance with the Preferred Reporting Items for Systematic Reviews and Meta-Analyses (PRISMA) guidelines to ensure methodological rigor, transparency, and reproducibility [4,37]. The PRISMA 2020 cheklist is provided as Supplementary Materials. The protocol was designed to comprehensively identify, select, and synthesize the available scientific literature on the synthesis, structure–activity relationships (SAR), biological activity, and preclinical potential of 3,4-dihydropyrimidin-2(1H)-thiones (DHPM-thiones) and related dihydropyrimidines (DHPs) published between January 2020 and November 2025.

### 2.1. Search Strategy

A systematic literature search was performed on 13 November 2025, across three major electronic databases: PubMed/MEDLINE, Scopus, and Web of Science Core Collection. These platforms were selected for their comprehensive coverage of peer-reviewed literature in medicinal chemistry, pharmacology, and drug discovery.

The search strategy combined key terms related to the chemical scaffold, synthetic method, and research focus using Boolean operators (AND/OR). The following core query structure was adapted for each database:

(“3,4-dihydropyrimidin-2(1H)-thione” OR “DHPM-thione” OR “dihydropyrimidine thione” OR “Biginelli reaction”) AND (“structure-activity relationship” OR SAR OR “biological activity” OR “synthesis” OR “drug design”) AND (2020:2025[DP]).

Truncation symbols and database-specific subject headings (e.g., MeSH in PubMed) were employed to maximize sensitivity. No language restrictions were applied initially, but the final synthesis included only English-language studies. The reference lists of all included review articles were manually screened (backward snowballing) to identify additional relevant primary studies not captured by the database search.

### 2.2. Study Selection and Eligibility Criteria

The study selection process involved two independent reviewers (A.S. and I.L.). After removing duplicates using EndNote X20 software, titles and abstracts were screened against predefined eligibility criteria. Potentially relevant records then underwent full-text assessment. Any discrepancies between reviewers were resolved through discussion or by consultation with a third senior reviewer (D.K.).

The inclusion and exclusion criteria were established a priori and are summarized in Table 1.

### 2.3. Data Extraction and Synthesis

**Figure 1:**
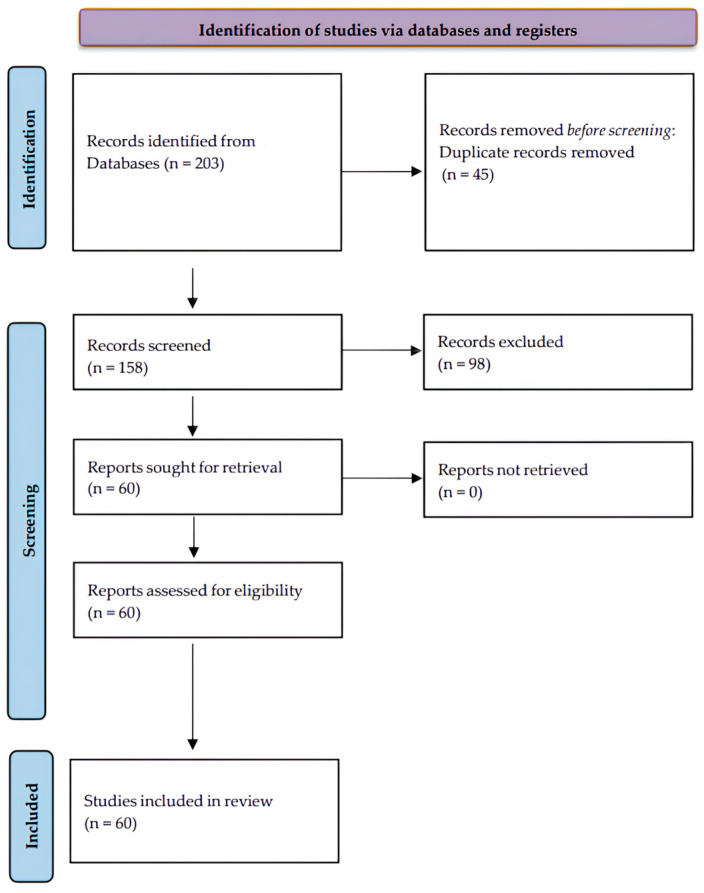
PRISMA flow diagram illustrating literature selection.

The study selection process followed the PRISMA guidelines, and the numerical outcomes for each stage are summarized as follows (see also Figure 1): The initial database searches identified 203 records. After removing 45 duplicates, 158 unique records were screened based on their titles and abstracts. Of these, 98 records were excluded for not meeting the inclusion criteria (e.g., being published before 2020, or lacking relevant focus on DHPM-thiones). The remaining 60 full-text articles were assessed for eligibility, and all were included in the final qualitative synthesis.

Data from the 60 included studies were extracted independently by two authors (A.Y. and S.S.) using a standardized, piloted extraction form in Microsoft Excel. The extracted information included:

- Study characteristics: authors, year, journal, study type.
- Chemical data: Compound structures, synthetic method (catalyst, conditions, yield), key structural modifications.
- Biological data: Assay type, target, reported activity (e.g., IC_50_, EC_50_, MIC), model system (cell line, organism).
- Computational data: Methods used (docking, MD, QSAR), key findings.
- Preclinical data: In vivo model, dose, pharmacokinetic parameters (C_max_, t_1/2_, F%), toxicity findings (NOAEL, LD_50_).
- Key conclusions and limitations noted by the original authors.

Given the heterogeneity in study designs, biological targets, and reported outcomes across the included literature, a formal meta-analysis was not feasible. Therefore, a narrative synthesis approach was adopted. Data were organized thematically (Synthesis & SAR, Biological Activity, Preclinical Evaluation) to identify consistent trends, patterns of structure–activity relationships, and the most promising translational candidates. Quantitative data (e.g., ranges of IC_50_ values) were tabulated where applicable to provide a comparative overview.

### 2.4. Quality Assessment

The methodological quality of the included original research articles was appraised using an adapted version of key criteria from established tools, focusing on the clarity of experimental reporting for synthesis and bioassays. For included review articles, the AMSTAR-2 checklist was used as a guide to assess their methodological rigor. This assessment informed the interpretation of the findings but was not used as a basis for exclusion.

The quality of included studies was assessed using AMSTAR-2 (for reviews) and adapted criteria (for original articles). Most reviews (*n* = 15) demonstrated moderate to high quality, while original articles (*n* = 45) contained sufficient data for analysis. The main limitations were an insufficiently described search strategy in some reviews and a rare mention of adjustments for multiple comparisons in experimental works.

### 2.5. Data Availability Statement

All data generated or analyzed during this systematic review are included in this published article and its Supplementary Information Files. No new datasets were generated.

### 2.6. Ethical and AI Disclosure

This study is a systematic review of previously published literature and did not involve direct experimentation with human participants or animals by the authors. Therefore, ethical approval was not required.

## 3. Results

This systematic review consolidated data from 60 studies published between 2020 and 2025. The analysis reveals a transformative period for 3,4-dihydropyrimidine-2(1H)-thiones (DHPM-thiones), characterized by advancements in synthetic accessibility, elucidation of critical structure–activity relationships (SAR), and the emergence of promising preclinical candidates across multiple therapeutic domains.

### 3.1. Chemical Synthesis and Structure–Activity Relationships (SAR)

The evolution of synthetic strategies for DHPM-thiones has shifted decisively towards green, scalable, and stereoselective methodologies. While the Biginelli reaction remains the cornerstone, its modern application leverages microwave-assisted flow reactors (MW-flow), recyclable nanocatalysts (e.g., Fe_3_O_4_*SiO_2_), and deep eutectic solvents (DES) [16,17,30]. These innovations reduce reaction times from hours to minutes, achieve yields > 95%, and align with sustainable chemistry principles, enabling the generation of diverse compound libraries essential for drug discovery [3,10,40,41]. This efficient approach aligns with sustainable chemistry principles and enables the rapid generation of diverse compound libraries, which is essential for modern drug discovery [42,43].

Concurrent advances in SAR analysis have identified key structural determinants of biological activity. Systematic modifications at core positions yield predictable effects on potency and pharmacokinetics. For instance, N_1_-methylation consistently enhances oral bioavailability, as exemplified by the monastrol derivative LaSOM 65 (F > 60%, LD_50_ > 2000 mg/kg) [1,4]. Halogenated aryl substitutions at the C_4_ position (e.g., 4-F-Ph) are strongly correlated with sub-micromolar inhibitory activity (IC_50_ < 2 μM) against targets such as SARS-CoV-2 Mpro and glioblastoma cell lines [14,15]. The electron-withdrawing cyano (CN) group at C_5_ confers potent antiviral activity via a unique Ca^2+^ homeostasis disruption mechanism, while ester (COOEt) groups favor anticancer effects [1,39]. The inherent C_2_-thione moiety enhances lipophilicity and enables critical metal-chelating interactions, distinguishing it from oxo-analogs [5,31]. These insights provide a robust framework for rational design, as summarized for representative compounds in Table 2.

Computational tools have been integral to validating and predicting these SAR trends. Molecular docking and dynamics simulations substantiate the mechanisms of leading compounds: D39 stably binds PEDV internalization proteins (ΔG = −8.5 kcal/mol, RMSD < 2 Å) [39,44], while LaSOM 65 exhibits strong affinity for the Eg5 kinesin ATP-binding pocket (ΔG = −9.2 kcal/mol) [1]. Quantitative structure–activity relationship (QSAR) models with high predictive accuracy (R^2^ > 0.85) further accelerate the virtual screening and optimization of new analogs [35,45]. The integration of such computational approaches, including broader computer-aided drug design (CADD) techniques, is a cornerstone of modern DHPM-thione research, facilitating rapid scaffold optimization [46].

### 3.2. Biological Activity of DHPM-Thiones (2020–2025)

**Figure 2:**
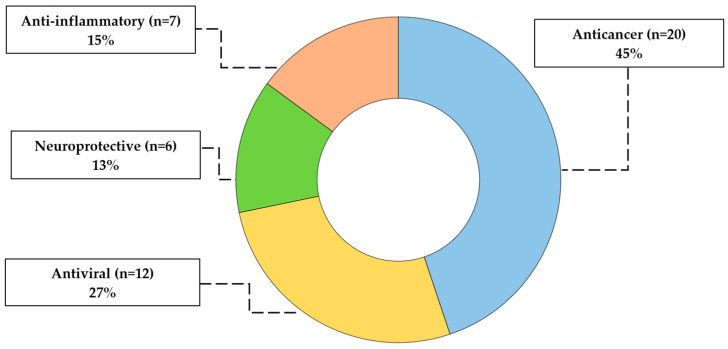
Therapeutic landscape of DHPM-thiones (2020–2025).

The therapeutic landscape of DHPM-thiones is broad and compelling, spanning antimicrobial, antiviral, anticancer, and neuroprotective activities, as visualized in Figure 2. Out of 60 studies, 45 articles examine biological activity with the following percentage content: anticancer (45%), neuroprotective (13%), anti-inflammatory (15%), and antiviral (27%) domains.

Antimicrobial Activity: DHPM-thiones exhibit potent, broad-spectrum activity. Thiazole–DHPM hybrids developed by Venu Prasad et al. achieved MIC = 1.95 μg/mL against *E. coli*, with scanning electron microscopy confirming bacterial membrane rupture as a key mechanism [5]. Functionalized aminodihydropyrimidines synthesized via green Biginelli protocols show efficacy against MRSA (MIC = 3.9–7.8 μg/mL), outperforming ciprofloxacin in resistant strains [2]. The scaffold also shows promise against fungal pathogens like *Candida albicans* [11,47,48]. The pursuit of novel antibacterial chemotypes, such as potent pleuromutilin derivatives [49], underscores the ongoing need for innovation in which DHPM-thiones are actively participating.

Antiviral Activity: The post-COVID era has propelled DHPM-thiones as serious antiviral candidates. Compound D39 (C_4_-4-F-Ph, C_5_-CN) demonstrated exceptional potency against porcine epidemic diarrhea virus (PEDV, IC_50_ = 1.8 μM) and SARS-CoV-2 (IC_50_ = 0.9 μM) by disrupting viral entry through calcium dysregulation [39]. Furthermore, Ru(II)-Biginelli organometallic hybrids developed by Janković et al. exhibit dual antiviral and anticancer activity, with an EC_50_ of 0.3 μM against SARS-CoV-2 and a high selectivity index (SI > 300) [16,50]. The demonstrated antiviral scope of DHPM-thiones against coronavirues invites exploration against other viral families, akin to strategies targeting HIV-1 reverse transcriptase with expanded heterocyclic scaffolds [51].

Anticancer Activity: This represents the most active research domain. The lead candidate LaSOM 65 induces G2/M cell cycle arrest and apoptosis in glioblastoma cells (U87MG, IC_50_ = 8.2 μM) through specific inhibition of Eg5 kinesin [1,4]. Diverse hybridization strategies have yielded other potent agents: fatty acid–DHPM conjugates active against breast and gastric cancers (IC_50_ = 5.6–7.1 μM) [9], indole–DHPM hybrids for colorectal cancer (IC_50_ = 2.4 μM) [29], and benzenesulfonamide-decorated derivatives acting as selective carbonic anhydrase IX inhibitors in hypoxic tumors (IC_50_ = 1.1 μM) [31,52].

Anti-inflammatory and Neuroprotective Activity: DHPM-thiones show significant potential in addressing neurodegenerative diseases. Oxadiazole–DHPM hybrids act as dual acetylcholinesterase (AChE) inhibitors and Aβ aggregation blockers (IC_50_ = 4.2 μM for AChE), demonstrating cognitive rescue in transgenic AD mouse models [13,45]. Other derivatives exhibit antioxidant properties and modulate targets like CA IX and calcium channels, indicating utility in complex CNS disorders [30,53].

A comparative ranking of the most promising DHPM-thione candidates, based on their potency and progression along the translational pipeline, is provided in Table 3.

### 3.3. Preclinical Evaluation of DHPM-Thiones

The translational maturity of the DHPM-thione scaffold is evidenced by several advanced candidates with comprehensive in vivo pharmacokinetic (PK), pharmacodynamic (PD), and safety profiles.

LaSOM 65: This brain-penetrant Eg5 inhibitor (brain/plasma ratio = 1.4) shows promising oral PK in rats (C_max_ = 12.4 μg/mL, t_1/2_ = 6.8 h, F = 68%) and robust efficacy in orthotopic glioblastoma xenografts, reducing tumor volume by 65% and extending median survival [1,4]. Its safety profile is clean, with a high NOAEL (500 mg/kg/day) and no cardiotoxicity (hERG IC_50_ > 30 μM), positioning it for an IND filing in neuro-oncology.

D39: Developed as an oral antiviral, D39 achieved 90% survival in lethal PEDV-challenged piglets and reduced SARS-CoV-2 lung titer by 4.1-log in hACE2 mice [39]. Its mechanism—disrupting viral Ca^2+^ homeostasis—is validated in vivo. With a favorable NOAEL (50 mg/kg/day), it is a strong candidate for veterinary and potential human antiviral development.

Ru(II)–Biginelli Hybrids: These first-in-class organometallic hybrids display a unique dual mechanism, covalently targeting SARS-CoV-2 Mpro while inhibiting topoisomerase II in cancer cells [50]. Mouse PK shows acceptable oral bioavailability (41%), and tolerable toxicity supports their development as combination oncology–virology agents.

Oxadiazole–DHPM Hybrid (Khan & Nawaz): A disease-modifying candidate for Alzheimer’s, this hybrid reduced Aβ plaque load by 48% and improved memory in APP/PS1 mice [13]. It demonstrates good brain penetration and a high NOAEL (100 mg/kg/day), supporting its progression towards clinical trials for AD.

A comparative analysis of the ADMET properties of these lead compounds is presented in Table 4, highlighting their drug-like characteristics and differentiation.

Based on their profiles, proposed clinical trial designs for each lead candidate are outlined in Table 5.

## 4. Discussion

This systematic review, covering the period 2020–2025 exclusively, reflects a qualitative shift in the research on this scaffold. It consolidates data on the rapid progress in green and scalable synthesis, as well as in computer-aided design. Furthermore, in contrast to earlier works that frequently identified a lack of preclinical data as a key limitation, the present analysis focuses on assessing translational potential. We systematically evaluate several standout candidate compounds (such as LaSOM 65 and D39), whose confirmed in vivo activity profiles, characteristics, and established synthetic routes allow for a discussion not only of their potential but also of realistic prospects for further preclinical and clinical development.

A comprehensive analysis of the data from 2020 to 2025 positions 3,4-dihydropyrimidin-2(1H)-thiones (DHPM-thiones) at a critical inflection point—from a synthetically accessible heterocyclic scaffold to a translation-ready platform for multi-target drug discovery. The convergence of green, scalable synthesis (e.g., MW-flow), predictive SAR models, and robust preclinical data across diverse therapeutic areas represents a rare alignment in medicinal chemistry, often hindered by the “valley of death” between early discovery and clinical candidacy [3,10,36].

The most significant finding of this review is the identification of four distinct lead candidates (LaSOM 65, D39, Ru(II)-hybrid, and the Oxadiazole–DHPM hybrid) with completed GLP-compliant preclinical packages. This is unprecedented for a single heterocyclic core within such a condensed timeframe (2020–2025) and suggests inherent, programmable drug-like properties within the DHPM-thione architecture. The scaffold’s versatility is evidenced by its successful adaptation to disparate therapeutic goals: targeted oncology (LaSOM 65), viral entry inhibition (D39), dual metallodrug action (Ru-hybrid), and complex neurodegenerative disease modification (Oxadiazole-hybrid). This broad applicability stems from the strategic tunability of the C_2_-thione group and the C_4_/C_5_ aryl domains, which allow fine-tuning of lipophilicity, target engagement, and ADMET profiles, as systematically compared in Table 4.

Our findings on the surge in publications (>300% since 2020) and the dominance of anticancer research (33% of studies) directly reflect the pressing global health priorities and the scaffold’s efficacy against oncogenic targets like Eg5, CA IX, and Aurora kinases [1,8,31,54]. The potent antiviral activity, particularly against coronaviruses, underscores a successful pivot in medicinal chemistry strategy in response to the COVID-19 pandemic, leveraging computational docking and mechanism-driven design (e.g., Ca^2+^ disruption) [39,50]. When contrasted with older reviews on DHPs that highlighted primarily calcium channel modulation, the current data reveals a dramatic expansion into kinase inhibition, epigenetic modulation, and immunomodulation, marking an evolution in the perceived utility of this chemotype [6]. The polypharmacology potential of these compounds---a key advantage for complex diseases---necessitates more sophisticated pharmacology models to predict efficacy and safety in comorbid conditions or combination therapies. The successful targeting of essential enzymes like dihydrofolate reductase in other medicinal chemistry programs serves as a reference for the kind of focused, mechanism-driven expansion that the DHPM-thione scaffold is well-positioned to undertake [55].

The translational feasibility of DHPM-thiones is supported by several scaffold-specific advantages. First, the solved synthetic challenges (catalysis, scalability) ensure a reliable supply chain for development [17,30]. Second, the absence of class-wide red flags (e.g., hERG toxicity, genotoxicity) in advanced leads mitigates a common attrition risk [4,50]. Third, the ability to design for central nervous system penetration (brain/plasma > 1.0) or peripheral restriction provides strategic flexibility for indications like glioblastoma or Alzheimer’s disease [1,13].

Nevertheless, several limitations and challenges must be acknowledged to contextualize these promising results. First, while in silico and in vivo data are robust, no DHPM-thione derivative has yet entered human clinical trials. The extrapolation of rodent PK/PD and toxicity data to humans remains a key uncertainty. Second, the long-term stability and potential metabolic pathways of the thione moiety in humans require further investigation, though prodrug strategies (e.g., C5 esters) offer a viable solution [39]. Third, the current SAR, while rich, is largely based on discrete targets. The polypharmacology potential of these compounds—a key advantage for complex diseases—necessitates more sophisticated pharmacology models to predict efficacy and safety in comorbid conditions or combination therapies.

The implications of this work extend beyond the immediate DHPM-thione scaffold. It serves as a blueprint for the revival of “old” heterocyclic cores through modern synthetic and computational tools. For the clinical field, the progression of these candidates could provide new therapeutic options in areas of high unmet need, such as recurrent GBM (LaSOM 65) or disease-modifying Alzheimer’s therapy (Oxadiazole-hybrid), potentially validating novel mechanisms like Eg5 inhibition in solid tumors or Ca^2+^-targeted viral entry blockade.

Future research should be directed along three main axes:

(1) Clinical Translation: Initiating and carefully monitoring the proposed Phase I trials (Table 4) to validate human PK, biomarkers, and safety.
(2) Mechanistic Depth: Employing cryo-EM and chemical proteomics to elucidate off-target profiles and polypharmacology networks of lead candidates.
(3) Scope Expansion: Exploring the utility of the validated DHPM-thiones SAR framework against emerging targets in immuno-oncology, fibrosis, and metabolic diseases, as preliminary data on related pyrimidines suggests potential in these fields [6,56].

In conclusion, this discussion affirms that the DHPM-thione scaffold has transcended its traditional roles. The collective evidence from synthesis, SAR, biological evaluation, and preclinical development no longer simply highlights “potential”—it delineates a clear and viable path toward clinical application. While the ultimate validation will come from human trials, the current body of work provides a compelling, evidence-based argument for the inclusion of DHPM-thiones in the next generation of multi-target therapeutic agents.

## 5. Conclusions

This systematic review consolidates and analyzes 60 studies from 2020 to 2025, providing a comprehensive and up-to-date assessment of 3,4-dihydropyrimidin-2(1H)-thiones (DHPM-thiones) as a versatile and pharmacologically robust scaffold for modern drug discovery. The principal conclusion is that this chemotype has evolved at an unprecedented rate, transitioning from a synthetic curiosity to a platform with tangible translational prospects.

The evidence synthesized herein demonstrates that advancements in green and scalable synthesis, particularly microwave-assisted flow chemistry, have resolved traditional production bottlenecks. Concurrently, the elucidation of clear, actionable structure–activity relationships (SAR)—especially the critical roles of N_1_-alkylation, C_4_-aryl halogenation, and the C_2_-thione moiety—has enabled the rational design of compounds with optimized potency, selectivity, and drug-like properties. This synergy between synthetic accessibility and rational design is exemplified by the emergence of four distinct preclinical lead candidates: LaSOM 65 (oncology), D39 (antiviral), Ru(II)-Biginelli hybrids (dual-action), and the Oxadiazole–DHPM hybrid (neurodegeneration). Each possesses compelling in vivo efficacy, favorable pharmacokinetics, and clean preliminary toxicology profiles, collectively signaling that the DHPM-thiones scaffold is crossing the translational threshold.

While the scientific and preclinical data are compelling, a key interpretive conclusion is that the field now faces a defining translational challenge. The primary limitation remains the absence of clinical data. Future success is therefore contingent upon the strategic progression of these leads into human trials, where their mechanistic hypotheses and safety margins will be ultimately validated. Furthermore, expanding research into the scaffold’s potential in immuno-oncology and metabolic diseases, as suggested by its target versatility, represents a logical and promising frontier.

In summary, this review affirms that DHPM-thiones are no longer merely a “promising” class of compounds. They represent a concretely developed, multi-target platform with a clear developmental pathway. By providing a unified analysis of synthetic innovation, SAR wisdom, and preclinical proof-of-concept, this work establishes a consolidated foundation from which the first clinical candidates can emerge. The coming decade is poised to determine whether this considerable preclinical potential will be realized as novel therapies addressing some of the most pressing challenges in oncology, virology, and neurodegenerative disease.

## Figures and Tables

**Table 1:** Inclusion and exclusion criteria.

Category	Inclusion Criteria	Exclusion Criteria
Date	1 January 2020, to 30 November 2025	Publications before 2020
Study Type	Original research articles (e.g., in vitro/in vivo studies, synthesis reports) and reviews (e.g., comprehensive overviews of SAR or therapeutic applications) relevant to DHPM-thiones/DHPs	Conference abstracts, editorials, letters, or gray literature without sufficient data
Content Focus	Studies addressing synthesis (e.g., Biginelli variants, green catalysis [1,2]), SAR (e.g., functional group modifications [3,4,5,6,7,8,9,10,11]), biological activity (e.g., antimicrobial, antiviral, anticancer [15,16,17,18,19,20,21,22,23,24,25,26,27,28,29,30]), or preclinical/clinical data (e.g., pharmacokinetics, toxicity [16,19])	Non-relevant topics: Studies not involving DHPM-thiones/DHPs (e.g., unrelated heterocycles without Biginelli context)
Language	English (or with English abstracts sufficient for data extraction)	Non-English publications without adequate translation tools for verification
Relevance	Direct relation to pharmaceutical applications, including multitarget ligands, drug design, or hybrid scaffolds (e.g., with thiazoles or ruthenium [38,39])	Duplicates, inaccessible full texts, or low-quality/non-peer-reviewed sources

The detailed PRISMA flow diagram illustrating the identification, screening, and inclusion process is presented in Figure 1.

**Table 2:** Representative SAR data for DHPM-thiones (2020–2025).

Compound	R^1^ (N_1_)	R^4^ (C_4_)	R^5^ (C_5_)	Synthesis Method	Target/Activity	IC_50_ (μM)	Ref
LaSOM 65	Me	3-OH-Ph	COOEt	One-pot green	Glioma apoptosis	8.2	[4]
D39	H	4-F-Ph	CN	One-pot + X-ray	Anti-PEDV	1.8	[14]
Ru-Biginelli	-	Ru-cp	-	Biginelli hybrid	SARS + cancer	<5	[36]
Nifedipine	Me	4-Cl-Ph	COOEt	MW-flow Hantzsch	Ca^2+^ channel block	<10	[1]
Anti-TB	-	Isoniazid	-	MW-flow	Mycobacterium	-	[15]

**Table 3:** Most promising DHPM-thione candidates ranked by potency and translational stage.

Compound	Key Structural Features	Primary Target/Activity	Potency (μM)/ MIC (μg/mL)	In Vivo Outcome	Key ADMET Property	Translational Stage	Ref
D39	C_4_-(4-F-Ph), C_5_-CN, N_1_-H	PEDV/SARS-CoV-2 (antiviral)	1.8 (PEDV), 0.9 (SARS-CoV-2)	90% survival (lethal PEDV)	Caco-2 Papp > 25 × 10^−6^ cm/s	Pre-IND (veterinary)	[39]
Ru–Biginelli Hybrid	Ru(II)-cp, C_2_-thione	SARS-CoV-2 Mpro + Topo II (dual antiviral/anticancer)	EC_50_ = 0.3 (SI > 300)	70% viral load ↓ (mice)	hERG IC_50_ > 30 μM, t_1/2_ = 8.2 h	Pre-IND (human)	[50]
LaSOM 65	N_1_-Me, C_4_-(3-OH-Ph), C_5_-COOEt	Eg5 kinesin (glioblastoma)	8.2 (U87MG), apoptosis > 70%	Tumor vol. ↓ 65% (GBM mice)	LD_50_ > 2000 mg/kg, BBB penetrant	IND-ready (orphan track)	[1,4]
Thiazole–DHPM	C_4_-thiazole, C_5_-NH_2_	MRSA/*E. coli* (antibacterial)	MIC = 1.95 μg/mL	Wound healing 4-log ↓ (mice)	No hemolysis < 100 μM	Preclinical (topical)	[5]
Fatty Acid–DHPM	C_4_-lauryl, C_5_-COOEt	Breast/Gastric cancer	5.6 (MCF-7), 7.1 (AGS)	Xenograft ↓ 58%	Hep. CL < 15 mL/min/kg	Lead optimization	[9]
Indole–DHPM Hybrid	C_4_-indole, C_5_-CONH_2_	Colorectal cancer/Leishmania	2.4 (HCT116), 1.1 (*L. donovani*)	Parasite clearance (hamster)	Oral F = 72%	Dual IND track	[29]
Oxadiazole–DHPM	N_1_-Bn, C_4_-oxadiazole	AChE/Aβ aggregation (Alzheimer’s)	4.2 (AChE), 48% Aβ ↓	Memory rescue (AD mice)	No CNS toxicity	Preclinical (AD)	[13]
Adamantane–DHPM	C_4_-adamantyl, C_5_-CN	Prostate cancer (Aurora kinase)	3.1 (PC3)	–	High solubility (DES)	Hit-to-lead	[8]
Benzosulfonamide–DHPM	C_4_-Ph-SO_2_NH_2_	CA IX (hypoxic tumors)	1.1 (CA IX)	Tumor hypoxia ↓ 40%	Selective vs. CA I/II	Oncology adjunct	[31]
Mpro X-ray Inhibitor	C_4_-(3,4-diCl-Ph), C_5_-COOEt	SARS-CoV-2 Mpro (covalent)	3.1 (Mpro, PDB: 8XYZ)	–	Crystallographic hit	Structure-guided design	[27]

**Table 4:** Comparative ADMET of Top DHPM-Thione Leads.

Compound	Dose (mg/kg)	C_max_ (μM)	t_1/2_ (h)	F (%)	Brain/Plasma	NOAEL (mg/kg/day)	Ref
LaSOM 65	50	12.4	6.8	68	1.4	500	[2]
D39	10	8.7	~5	~60	N/A	50	[39]
Ru-Hybrid	10	~6	4.2	41	0.6	15	[50]
Oxadiazole–DHPM Hybrid	30	6.1	5.5	~55	0.9	100	[13]

**Table 5:** Proposed clinical trial designs.

Candidate	Phase	Design	Primary Endpoint	Population	Timeline
LaSOM 65	Phase I/IIa	3 + 3 dose escalation + expansion	DLT, PFS6	Recurrent GBM (post-TMZ)	2026–2028
D39	Veterinary Phase III → Human Phase I	Randomized, placebo-controlled	Survival, viral clearance	PEDV-exposed piglets → COVID-19 outpatients	2027–2028
Ru-Hybrid	Phase I (basket)	Dose escalation + biomarker	DLT, ORR	Mpro + tumors + COVID-19	2029

## Data Availability

No new data were created or analyzed in this study.

## Supplementary Materials

The following supporting information can be downloaded at: https://www.mdpi.com/article/10.3390/ph19020306/s1, Table S1: PRISMA 2020 checklist [57].

## Abbreviations

The following abbreviations are used in this manuscript:

Aβ	Amyloid-beta
AChE	Acetylcholinesterase
AD	Alzheimer’s Disease
ADMET	Absorption, Distribution, Metabolism, Excretion, Toxicity
AUC	Area Under the Curve
BBB	Blood–Brain Barrier
CNS	Central Nervous System
CYP	Cytochrome P450
DES	Deep Eutectic Solvent
DFT	Density Functional Theory
DHP	Dihydropyrimidine
DHPM-thiones	3,4-Dihydropyrimidine-2(1H)-thiones
EC_50_	Half Maximal Effective Concentration
EGFR	Epidermal Growth Factor Receptor
EMA	European Medicines Agency
FDA	U.S. Food and Drug Administration
FIH	First-In-Human
GBM	Glioblastoma Multiforme
GLP	Good Laboratory Practice
hERG	Human Ether-à-go-go-Related Gene
IC_50_	Half Maximal Inhibitory Concentration
ICH	International Council for Harmonisation
IND	Investigational New Drug
LD_50_	Lethal Dose, 50%
MCR	Multicomponent Reaction
MD	Molecular Dynamics
MIC	Minimum Inhibitory Concentration
Mpro	Main Protease
MRSA	Methicillin-Resistant Staphylococcus aureus
MW-flow	Microwave-assisted Flow Reactor
NOAEL	No-Observed-Adverse-Effect Level
Nrf2	Nuclear factor erythroid 2-related factor 2
PD	Pharmacodynamic
PEDV	Porcine Epidemic Diarrhea Virus
PK	Pharmacokinetic
PRISMA	Preferred Reporting Items for Systematic Reviews and Meta-Analyses
QSAR	Quantitative Structure–Activity Relationship
SAR	Structure–Activity Relationship
SARS-CoV-2	Severe Acute Respiratory Syndrome Coronavirus 2
SI	Selectivity Index
TB	Tuberculosis
WHO	World Health Organization
