# Supplementary material for: Structural and Functional Aspects of DHPM-Thiones and Their Derivatives: A Critical Review of Pharmaceutical Potential
Source: Pharmaceuticals (Basel). 2026 Feb 12;19(2):306. doi: 10.3390/ph19020306 (PMC12943597; doi:10.3390/ph19020306)
Supplement: Supplementary file 1 [file pharmaceuticals-19-00306-s001.zip › pharmaceuticals-4126877-supplementary.pdf]

PRISMA 2020 Checklist

Section and Topic	Item #	Checklist item	Location where item is reported
**TITLE**			
Title	1	Identify the report as a systematic review.	Title page: "Structural and functional aspects of DHPTS derivatives: a critical review of pharmaceutical potential" and article type "Systematic Review".
**ABSTRACT**			
Abstract	2	See the PRISMA 2020 for Abstracts checklist.	The abstract is structured with headings: Background/Objectives, Methods, Results, Conclusions.
**INTRODUCTION**			
Rationale	3	Describe the rationale for the review in the context of existing knowledge.	Section 1. Introduction: First paragraph describing global challenges (antimicrobial resistance, post-COVID gaps) and the promise of DHPM-thiones.
Objectives	4	Provide an explicit statement of the objective(s) or question(s) the review addresses.	Section 1. Introduction, final sentences: "...aims to consolidate recent advancements (2020-2025) and evaluate the synthetic innovation, structure-activity relationships (SAR), and preclinical potential of these compounds."
**METHODS**			
Eligibility criteria	5	Specify the inclusion and exclusion criteria for the review and how studies were grouped for the syntheses.	Section 4.2. Study Selection and Eligibility Criteria and Table 1: Inclusion and Exclusion Criteria.
Information sources	6	Specify all databases, registers, websites, organisations, reference lists and other sources searched or consulted to identify studies. Specify the date when each source was last searched or consulted.	Section 4.1. Search Strategy: "A systematic literature search was performed on

PRISMA 2020 Checklist

Section and Topic	Item #	Checklist item	Location where item is reported
			November 13, 2025... across PubMed/MEDLINE, Scopus, and Web of Science Core Collection."
Search strategy	7	Present the full search strategies for all databases, registers and websites, including any filters and limits used.	Section 4.1. Search Strategy: Provides the core query structure with Boolean operators, terms, and date filters.
Selection process	8	Specify the methods used to decide whether a study met the inclusion criteria of the review, including how many reviewers screened each record and each report retrieved, whether they worked independently, and if applicable, details of automation tools used in the process.	Section 4.2. Study Selection...: "The study selection process involved two independent reviewers discrepancies resolved by discussion or a third reviewer (D.K.)."
Data collection process	9	Specify the methods used to collect data from reports, including how many reviewers collected data from each report, whether they worked independently, any processes for obtaining or confirming data from study investigators, and if applicable, details of automation tools used in the process.	Section 4.3. Data Extraction and Synthesis: "Data... were extracted independently by two authors using a standardized extraction form."
Data items	10a	List and define all outcomes for which data were sought. Specify whether all results that were compatible with each outcome domain in each study were sought (e.g. for all measures, time points, analyses), and if not, the methods used to decide which results to collect.	Section 4.3. Data Extraction...: Lists 6 categories of extracted data (study characteristics, chemical data, biological data, etc.).
	10b	List and define all other variables for which data were sought (e.g. participant and intervention characteristics, funding sources). Describe any assumptions made about any missing or unclear information.	Section 4.3. Data Extraction...: Same as above; includes study design, compound details, assay types, PK parameters.
Study risk of bias assessment	11	Specify the methods used to assess risk of bias in the included studies, including details of the tool(s) used, how many reviewers assessed each study and whether they worked independently, and if applicable, details of automation tools used in the process.	Section 4.4. Quality Assessment: "The methodological quality... was appraised using an adapted

Section and Topic	Item #	Checklist item	Location where item is reported
			version of key criteria... For reviews, the AMSTAR-2 checklist was used as a guide."
Effect measures	12	Specify for each outcome the effect measure(s) (e.g. risk ratio, mean difference) used in the synthesis or presentation of results.	Throughout Sections 2.1-2.3; primary measures are IC_{50}, EC_{50}, MIC (μM, μg/mL), and PK parameters (Cmax, t½, F%).
Synthesis methods	13a	Describe the processes used to decide which studies were eligible for each synthesis (e.g. tabulating the study intervention characteristics and comparing against the planned groups for each synthesis (item #5)).	Section 4.3. Data Extraction and Synthesis: "Data were organized thematically (Synthesis & SAR, Biological Activity, Preclinical Evaluation)..."
	13b	Describe any methods required to prepare the data for presentation or synthesis, such as handling of missing summary statistics, or data conversions.	Section 4.3. Data Extraction and Synthesis: "Quantitative data (e.g., ranges of IC_{50} values) were tabulated where applicable..."
	13c	Describe any methods used to tabulate or visually display results of individual studies and syntheses.	Sections 2.1-2.3 (Results): Data presented in narrative text, Tables 2-5, and Figure 2.
	13d	Describe any methods used to synthesize results and provide a rationale for the choice(s). If meta-analysis was performed, describe the model(s), method(s) to identify the presence and extent of statistical heterogeneity, and software package(s) used.	Section 4.3. Data Extraction and Synthesis: "Given the heterogeneity... a formal meta-analysis was not feasible. Therefore, a narrative synthesis approach was adopted."
	13e	Describe any methods used to explore possible causes of heterogeneity among study results (e.g. subgroup analysis, meta-regression).	Not applicable (narrative synthesis, no meta-analysis).
	13f	Describe any sensitivity analyses conducted to assess robustness of the synthesized results.	Not applicable.

PRISMA 2020 Checklist

Section and Topic	Item #	Checklist item	Location where item is reported
Reporting bias assessment	14	Describe any methods used to assess risk of bias due to missing results in a synthesis (arising from reporting biases).	Not explicitly described. Mention of checking reference lists in Section 4.1 to mitigate publication bias.
Certainty assessment	15	Describe any methods used to assess certainty (or confidence) in the body of evidence for an outcome.	Not applicable (GRADE assessment not performed). Quality assessment is described in Section 4.4.
**RESULTS**			
Study selection	16a	Describe the results of the search and selection process, from the number of records identified in the search to the number of studies included in the review, ideally using a flow diagram.	Sections 4.1 & 4.2: Numbers provided: 203 records, 45 duplicates removed, 158 screened, 98 excluded, 60 included. See Figure 1 (PRISMA flow diagram).
	16b	Cite studies that might appear to meet the inclusion criteria, but which were excluded, and explain why they were excluded.	Not provided for individual studies. General exclusion reasons are stated in Section 4.2 (duplicates, pre-2020, irrelevant).
Study characteristics	17	Cite each included study and present its characteristics.	The 60 included studies are cited in the References. Their characteristics are presented thematically in Sections 2.1-2.3 and summarized in Tables 2-5.
Risk of bias in studies	18	Present assessments of risk of bias for each included study.	Not presented for individual studies. Overall discussion of evidence limitations is in Sections 3 (Discussion) and 5 (Conclusions).
Results of individual studies	19	For all outcomes, present, for each study: (a) summary statistics for each group (where appropriate) and (b) an effect estimate and its precision (e.g. confidence/credible interval), ideally using structured tables or plots.	Results are synthesized thematically in Sections 2.1, 2.2, 2.3. Key

PRISMA 2020 Checklist

Section and Topic	Item #	Checklist item	Location where item is reported
			quantitative results for specific compounds are presented in the text and Tables 2, 3, 4.
Results of syntheses	20a	For each synthesis, briefly summarise the characteristics and risk of bias among contributing studies.	Sections 2.1, 2.2, 2.3 begin with a summary of the synthesized evidence for each theme.
	20b	Present results of all statistical syntheses conducted. If meta-analysis was done, present for each the summary estimate and its precision (e.g. confidence/credible interval) and measures of statistical heterogeneity. If comparing groups, describe the direction of the effect.	Not applicable (no meta-analysis).
	20c	Present results of all investigations of possible causes of heterogeneity among study results.	Not applicable.
	20d	Present results of all sensitivity analyses conducted to assess the robustness of the synthesized results.	Not applicable.
Reporting biases	21	Present assessments of risk of bias due to missing results (arising from reporting biases) for each synthesis assessed.	Not presented.
Certainty of evidence	22	Present assessments of certainty (or confidence) in the body of evidence for each outcome assessed.	Not presented (GRADE not done). Overall certainty is discussed in Sections 3 and 5.
**DISCUSSION**			
Discussion	23a	Provide a general interpretation of the results in the context of other evidence.	Section 3. Discussion: Paragraphs beginning with "The most significant finding...", "Our findings on the surge in publications...", "When contrasted with older reviews...".
	23b	Discuss any limitations of the evidence included in the review.	Section 3. Discussion: "However, several limitations and challenges must be acknowledged..." lists translational gaps, metabolic stability, etc.
	23c	Discuss any limitations of the review processes used.	Section 3. Discussion: Partially covered under evidence limitations (e.g., extrapolation from animal models).
	23d	Discuss implications of the results for practice, policy, and future research.	Section 3. Discussion: "The implications of this

Section and Topic	Item #	Checklist item	Location where item is reported
			work...", "Future research should be directed along three main axes...".
**OTHER INFORMATION**			
Registration and protocol	24a	Provide registration information for the review, including register name and registration number, or state that the review was not registered.	The review was not registered. Can be stated as: "This systematic review was not registered."
	24b	Indicate where the review protocol can be accessed, or state that a protocol was not prepared.	A protocol was not prepared separately. The methods are detailed in Section 4 (Materials and Methods).
	24c	Describe and explain any amendments to information provided at registration or in the protocol.	Not applicable.
Support	25	Describe sources of financial or non-financial support for the review, and the role of the funders or sponsors in the review.	Acknowledgments and Funding sections: "This research received no external funding." Acknowledges university support.
Competing interests	26	Declare any competing interests of review authors.	Conflicts of Interest section: "The authors declare no conflicts of interest."
Availability of data, code and other materials	27	Report which of the following are publicly available and where they can be found: template data collection forms; data extracted from included studies; data used for all analyses; analytic code; any other materials used in the review.	Data Availability Statement: "All data generated or analyzed during this systematic review are included in this published article... No new datasets were generated."

From: Page MJ, McKenzie JE, Bossuyt PM, Boutron I, Hoffmann TC, Mulrow CD, et al. The PRISMA 2020 statement: an updated guideline for reporting systematic reviews. BMJ 2021;372:n71. doi: 10.1136/bmj.n71. This work is licensed under CC BY 4.0. To view a copy of this license, visit https://creativecommons.org/licenses/by/4.0/
